# Topical Application of Oxylipin (3*S*)-16,17-Didehydrofalcarinol in Mice Infected with *Leishmania mexicana*: A Possible Treatment for Localized Cutaneous Leishmaniasis

**DOI:** 10.1021/acs.jnatprod.4c01411

**Published:** 2025-04-03

**Authors:** Ana G. Carrillo-Aké, José Delgado-Domínguez, Rocely Buenaventura Cervantes-Sarabia, Adriana Ruiz-Remigio, Jaime Zamora-Chimal, Norma Salaiza-Suazo, Luis W. Torres-Tapia, Sergio R. Peraza-Sánchez, Ingeborg Becker

**Affiliations:** † Unidad de Medicina Experimental, Facultad de Medicina, Universidad Nacional Autónoma de México (UNAM), Hospital General de México Dr. Balmis 148, Ciudad de México 06720, Mexico; ‡ Centro de Investigación Científica de Yucatán (CICY), Unidad de Biotecnología, Calle 43 #130, Col. Chuburná de Hidalgo, Mérida, Yucatán 97205, Mexico

## Abstract

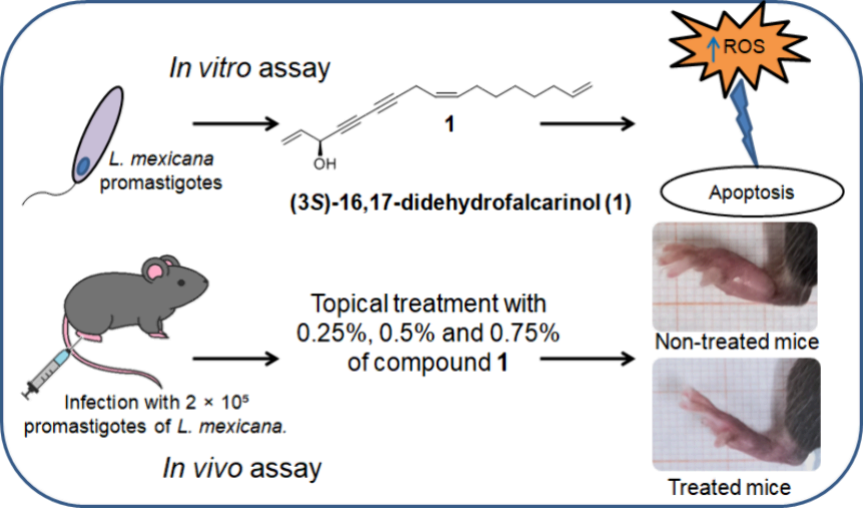

Pentavalent antimonials are the first-line treatment for localized cutaneous leishmaniasis. However, they have disadvantages such as their elevated toxicity, high costs, and parenteral application. Plant-derived compounds may be an alternative treatment against this disease. Previous *in vitro* studies have shown that (3*S*)-16,17-didehydrofalcarinol (**1**), a polyacetylene oxylipin isolated from *Tridax procumbens*, is active against *Leishmania mexicana*. We have analyzed the mechanism of action of compound **1**, evaluating reactive oxygen species production, apoptosis of *L. mexicana*, cytotoxicity in murine macrophages, and its efficacy in controlling the disease progression and parasite load when applied topically in C57BL/6 mice infected with *L. mexicana*. Results show that parasites incubated with 1.6 μM compound **1** significantly increased reactive oxygen species production (*p* ≤ 0.05). The percentage of apoptosis also increased significantly (*p* ≤ 0.05) and did not affect the viability of macrophages. The application of the topical formulations with 0.5% and 0.75% compound **1** for 7 weeks reduced disease progression and parasite load. We demonstrate that compound **1** generates the death of *L. mexicana* by apoptosis through reactive oxygen species production. We conclude that compound **1** can be used a possible alternative treatment for localized cutaneous leishmaniasis, enabling a less painful and more accessible therapy.

Leishmaniasis comprises a group of diseases caused by protozoan parasites of the genus *Leishmania* that can affect the skin, mucous membranes, and some internal organs. Currently, these diseases are present in more than 102 tropical and subtropical countries, mainly affecting people with limited resources.^1^ In Mexico, 99% of leishmaniasis cases that are registered each year correspond to the clinical picture of the localized cutaneous leishmaniasis (LCL), also known as “chiclero’s ulcer”, and is mainly caused by the species *Leishmania mexicana*.^2^ This clinical form is characterized by a small nodule that evolves to generate an ulcer at the site of the vector bite. These lesions can lead to skin mutilations and social stigmatization when it affects the face.^3^ Pentavalent antimonials such as Glucantime and Pentostam continue to be the main drugs to treat this disease; however, these compounds are highly toxic, their administration is intramuscular or intralesional and they are difficult to acquire due to their high cost.^4^

Several studies have shown that plants are an important source of bioactive compounds against various infectious agents such as bacteria, viruses, and parasites, and they could be used in the development of new, less toxic and less painful treatments to treat LCL.^5^ Examples of these compounds are polyacetylenic oxylipins that can be found in various plants of the Apiaceae, Araliaceae, and Asteraceae families and are characterized by having more than one carbon–carbon triple bond in their structure. They are produced from the oxidation of unsaturated fatty acids in all aerobic organisms. In plants, these compounds are biosynthesized from the dehydrogenation of oleic acid and linoleic acid leading to precursors such as crepenynic acid and dehydrocrepenynic acid. Subsequently, by additional dehydrogenation, oxidation or β-oxidation and/or α-oxidation reactions, and polyacetylenes of various chain lengths ranging from 10 to 18 carbon atoms, are produced.^6^ In general, C_17_ polyacetylenes display antitumoral, anti-inflammatory, antibacterial, antifungal, and antiviral properties, which is why they can be considered optimal compounds for the development of various drugs.^7^

Compound **1**, (3*S*)-16,17-didehydrofalcarinol, is a C_17_ polyacetylenic oxylipin isolated from the rhizomes of *Tridax procumbens* L. (Asteraceae), a plant weed that is used topically for the treatment of leishmaniasis ulcers in Guatemala.^8^ Previous studies have shown that this compound exerts a potent effect *in vitro* against *L. mexicana* parasites.^9^ However, its mechanism of action and its effect on the evolution of the disease caused by *L. mexicana* have not yet been described.
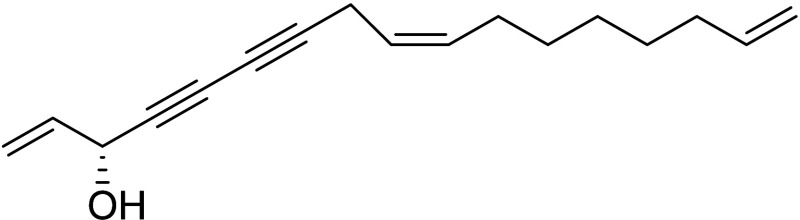


(3*S*)-16,17-didehydrofalcarinol (**1**)

Therefore, this work aims to establish the possible mechanism of action of compound **1** against *L. mexicana* promastigotes and to evaluate its topical efficacy for the treatment of Leishmania infections in a mouse model.

## Results and Discussion

**Figure 1:**
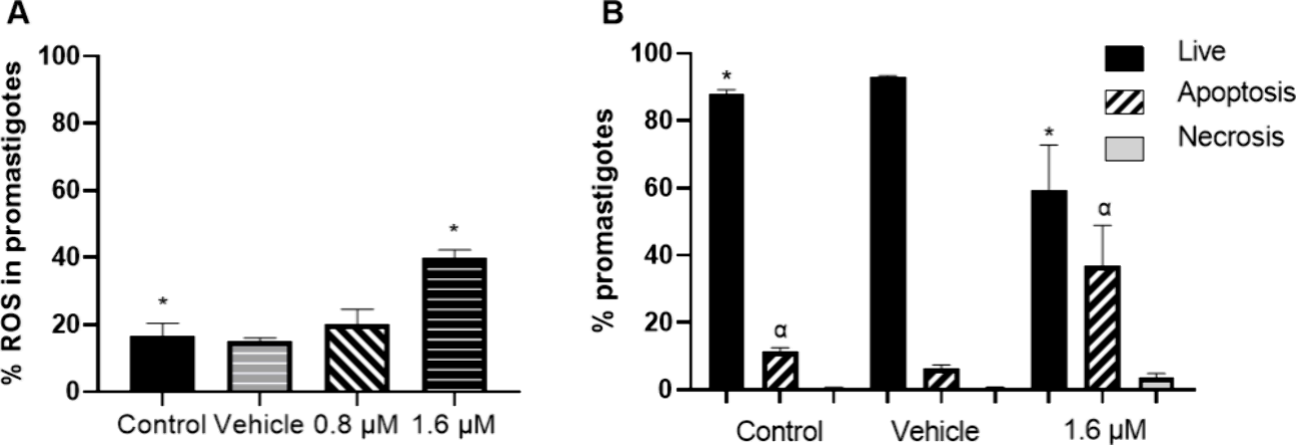
(A) Percentage of ROS in promastigotes incubated with 0.8 and 1.6 μM compound **1** for 72 h. Symbols indicate significant differences with respect to the nontreated control (*p* ≤ 0.05). (B) Percentage of viability, apoptosis, and necrosis in promastigotes treated with 1.6 μM compound **1** for 72 h. Symbols represent significant differences between groups (*p* ≤ 0.05). All data represent the mean ± SD of four independent experiments.

The results showed that reactive oxygen species (ROS) production in parasites incubated with 1.6 μM of compound **1** increased more than 2-fold (40%), compared to the percentage of ROS (16.5%) produced in nontreated parasites (*p* ≤ 0.05). The vehicle and the concentration of 0.8 μM did not generate a significant increase in ROS production with respect to the control (Figure 1A).

Subsequently, we evaluated the cell death process in *L. mexicana* promastigotes incubated with 1.6 μM of compound **1** for 72 h, using the markers Annexin V to measure apoptosis and 7-ADD to measure necrosis. The results showed that the percentage of live parasites decreased by 32.4% compared to the percentage of live parasites in the nontreated control (*p* ≤ 0.05). The percentage of apoptosis in parasites exposed to compound **1** increased significantly (36.9%) in relation to nontreated parasites, where only 11.4% of apoptotic parasites were found (*p* ≤ 0.05). Regarding the percentage of necrotic cells, no significant increase was observed when parasites were exposed to compound **1** compared to the control without treatment. Furthermore, the vehicle did not affect the viability of the parasites (Figure 1B).

ROS intermediates, such as superoxide anion (O_2_®), hydroxyl radical (−OH), and hydrogen peroxide (H_2_O_2_) are highly reactive molecules that originate in low concentrations during respiration, photosynthesis, and growth processes in plants, animals, and microorganisms. However, a significant increase in these molecules generates oxidative damage that can trigger apoptosis in cells.^10^ When 1.6 μM of compound **1** was added to the *L. mexicana* promastigote culture, a correlation between the increase in ROS and the increase in cell death by apoptosis was observed. This same effect has also been seen in other studies where some natural compounds have been evaluated against different species of *Leishmania*, such as betulin evaluated against promastigotes of *L. donovani* and carajurin against promastigotes of *L. amazonesis*.^11,12^ Although there are no reports confirming that either compound **1** or other polyacetylenic oxylipins induce apoptosis in *Leishmania* through the production of ROS, there are studies that propose that these fatty acids, having two or more triple bonds in their structure, act as alkylating molecules capable of trapping thiols by direct nucleophilic addition. This allows them to covalently bind to proteins or other biomolecules generating oxidative stress, as shown by a study where falcarinol (a C_17_ polyacetylenic oxylipin) binds covalently to cysteine in enzymes, such as mitochondrial aldehyde dehydrogenase (ALDH) in cancer cells, decreasing their activity, which can lead to oxidative stress and endoplasmic reticulum (ER) stress, causing cell damage, cell cycle arrest, and apoptosis.^13^ Therefore, this would be the first report that compound **1** induces an increase in ROS production in *L. mexicana* promastigotes, leading to cell death by apoptosis.

The process of death by apoptosis in mammalian cells has been described to occur through two pathways. The intrinsic apoptotic pathway that involves a signaling mechanism in the mitochondria, mainly by the activation of the pro-apoptotic members of the BCL-2 BAX and BAK family, facilitating the release of cytochrome C from the mitochondria and the subsequent activation of caspase-9, which activates other effector caspases such as caspases 3 and 7, generating apoptosis. And the extrinsic apoptotic pathway, which is initiated through the binding of the ligand to death receptors of the tumor necrosis factor (TNF) superfamily, which includes Fas, TNFR1, DR3, DR4 (TRAIL-R1), DR5 (TRAIL-R2) and DR6. After ligand binding, the death domain recruits Fas-associated proteins with dead domain (FADD), forming a death-inducing complex that helps to recruit procaspase-8 that activates caspase 8 and subsequently leads to the activation of caspases 3 and 7.^14^ However, in *Leishmania* it is not fully established how apoptosis occurs, since the caspases or classical death receptors have not been identified. However, several proteins such as metacaspases have been found that fulfill these same functions, making this process possible in *Leishmania* parasites.^15^

**Figure 2:**
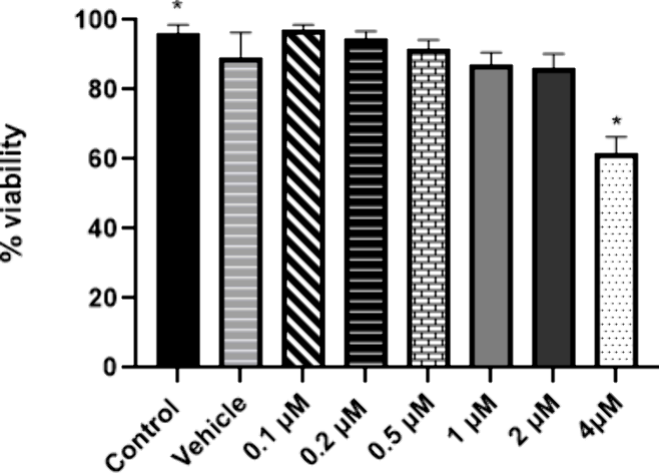
Effect of compound **1** on the viability of noninfected peritoneal macrophages. The viability of macrophages incubated with different concentrations of compound **1** for 48 h was assessed using the colorimetric XTT assay. Symbols indicate significant differences compared to the nontreated control (*p* ≤ 0.05). Bars represent the mean ± SD of three independent experiments.

After knowing the mechanism of action of compound **1**, we also examined whether it has a cytotoxic effect in murine peritoneal macrophages. Noninfected macrophages were incubated with different concentrations of compound **1** (0.1–4 μM) for 48 h. The viability of the cells was determined by the colorimetric assay with XTT. Only the highest concentration of compound **1** (4 μM) significantly reduced the viability of macrophages by 38.5%, with respect to the control without stimulus (*p* ≤ 0.05). The vehicle and the other concentrations did not affect the viability of macrophages (Figure 2).

The mean cytotoxic concentration (CC_50_) calculated for compound **1** was 1.5 ± 0.2 μg/mL. Subsequently, the selectivity index (SI) of compound **1** (SI = 5.5) was determined. The information obtained through SI of a compound is considered necessary for pharmacological research, since it provides guidance on its safety in relation to its efficacy. In our case it shows the safety of compound **1** on host cells (macrophages), compared to its toxicity on *Leishmania* parasites. Several studies indicate that plant-derived compounds with an SI > 5 are considered safe candidates for further preclinical research on leishmaniasis.^16^

After observing that the 1.6 μM dose of compound **1** did not generate a negative effect on the viability of intraperitoneal macrophages, we decided to explore whether this same concentration exerts an immunomodulatory effect on macrophages, since those cells are responsible for killing and controlling the number of parasites in the host as long as they are properly activated.^17^*Leishmania* promastigotes express surface molecules such as lipophosphoglycan (LPG), protease gp63, and acid phosphatases that are involved in the activation of macrophages by decreasing or increasing the production of microbicidal molecules such as nitric oxide (NO) and certain cytokines.^18^

**Figure 3:**
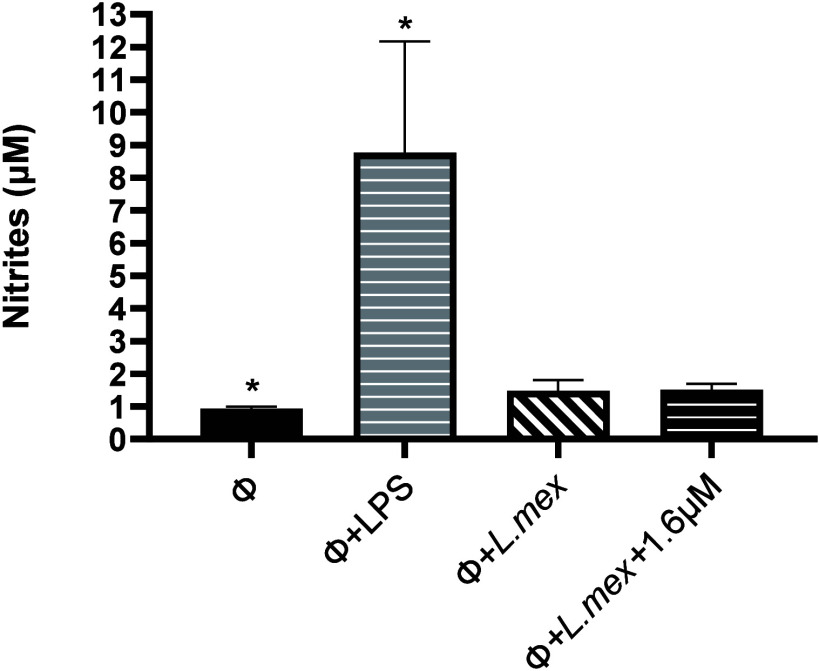
Effect of compound **1** on nitric oxide (NO) production in BMMs infected with *L. mexicana*. Nitrites (μM) were measured in nonstimulated macrophages (Φ), LPS-stimulated macrophages (Φ+LPS), nontreated *L. mexicana*-infected macrophages (Φ+*L.mex*) and infected treated macrophages with compound **1** (Φ+*L.mex*+1.6 μM) for 24 h. NO production was determined by Griess colorimetric assay. Symbols indicate statistically significant differences compared to nonstimulated macrophages (*p* ≤ 0.05). Data represent the mean ± SD of three independent experiments.

Because NO plays a primary role in the leishmanicidal activity of the macrophage, we evaluated whether incubation with compound **1** (1.6 μM) favors the production of this effector molecule in differentiated bone marrow-derived macrophages (BMMs) infected with *L. mexicana*. The results showed that compound **1** did not modify the NO production in *Leishmania mexicana* infected macrophages. As expected, LPS resulted in significantly higher NO production (*p* ≤ 0.05), compared to the basal control (Figure 3).

In general, secondary metabolites can act directly against the parasite, indirectly by stimulating the production of nitric oxide (NO) in macrophages, or by both pathways. For example, chalcones alters the structure of mitochondria resulting in inhibition of the respiratory chain; flavonoids inhibit DNA synthesis and induce ROS production triggering apoptosis; saponins decrease membrane potential causing loss of membrane integrity; alkaloids induce ROS and NO production; tannins increase NO production in infected macrophages and improve cytokine expression including IL-10, IL-12, TNF-α and IFN-γ; and terpenes increase NO production and induce apoptosis.^19^ Our current findings indicate that compound **1** does not stimulate NO production in BMMs infected with *L. mexicana*, which is consistent with the results obtained in a previous study.^9^ However, compound **1** does induce an increase in ROS, leading to apoptosis.

In the case of LCL, the cytokines produced by macrophages that favor the resolution of the disease are TNF alpha (TNF-α) and interleukin (IL) 12, which are known to have pro-inflammatory functions that participate in the activation of macrophages and the elimination of parasites.^20^ Other important cytokines produced by macrophages during LCL are IL-10 and transforming growth factor (TGF-β). These cytokines can contribute to the progression of the disease or aid in the resolution of the disease, which depends on the species of *Leishmania* and the host’s immune system.^21^ Therefore, we measured the production of TNF-α, IL-12 (p70), and IL-10 in (BMMs) infected with *L. mexicana* promastigotes and treated with 1.6 μM of compound **1** for 24 h. After incubation, the supernatants were collected and TNF-α, IL-12 (p70), and IL-10 concentrations were determined by a sandwich assay (ELISA).

**Figure 4:**
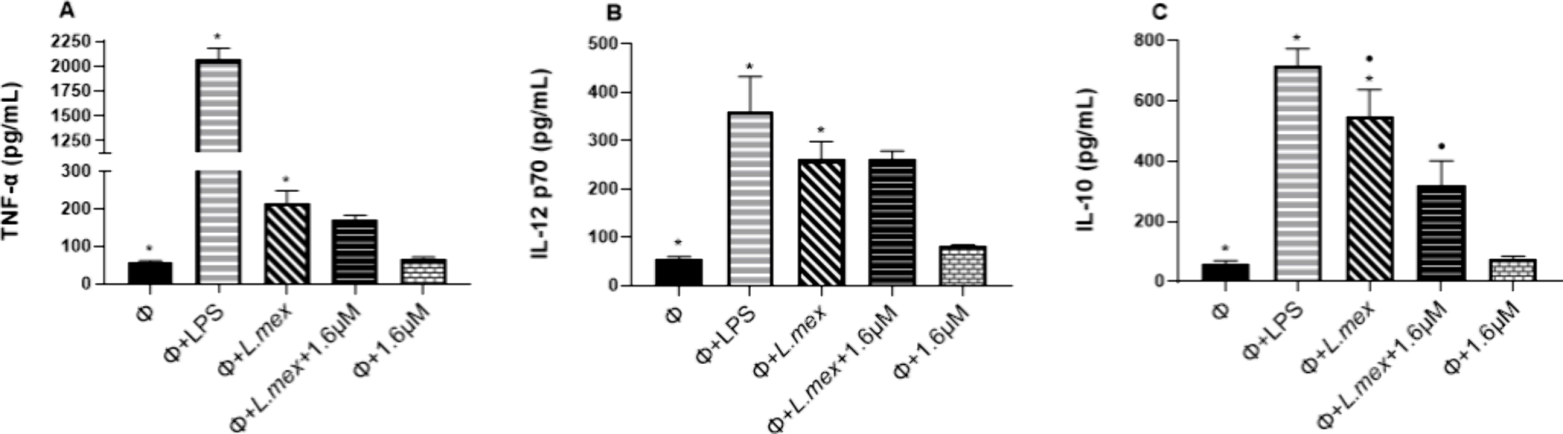
Effect of compound **1** on cytokine production in BMMs infected with *L. mexicana*. (A) TNF-α production. (B) IL-12 (p70) production. (C) IL-10 production. Nonstimulated macrophages (Φ), macrophages stimulated with LPS (Φ+LPS), nontreated *L. mexicana*-infected macrophages (Φ+*L.mex*), infected macrophages treated with compound **1** (Φ+*L.mex*+1.6 μM) and in macrophages stimulated with compound **1** (Φ+1.6 μM) for 24 h. Asterisks represent significant differences compared to nonstimulated macrophages. Circles represent significant differences compared to nontreated infected macrophages (*p* ≤ 0.05). Data represent mean ± SD of three independent experiments.

Figure 4A shows that TNF-α production in nontreated infected macrophages increased significantly to 216.3 pg/mL, compared to basal control (noninfected macrophages) (56.9 pg/mL) (*p* ≤ 0.05). In infected macrophages treated with compound **1**, TNF-α production decreased slightly without representing a significant difference compared to infected macrophages without treatment. IL-12 (p70) production in nontreated infected macrophages also increased significantly (260.7 pg/mL) compared to basal control (54.7 pg/mL) (*p* ≤ 0.05). The production of IL-12 in infected macrophages, treated with compound **1**, showed no significant differences compared to infected macrophages without treatment (Figure 4B). Regarding IL-10 production in infected and nontreated macrophages, the concentration of this cytokine increased 8.5-fold (547 pg/mL), compared to basal control (60.8 pg/mL), but decreased significantly by 41.8% (318.8 pg/mL) in infected macrophages treated with compound **1**, when compared to infected macrophages without treatment (*p* ≤ 0.05) (Figure 4C). The compound alone did not generate a significant increase in TNF-α, IL-12, and IL-10 compared to the basal control.

TNF-α plays a crucial role in *Leishmania* parasites elimination by activating macrophages to increase their phagocytic capacity.^22^ However, excessive production of this cytokine is associated with increased tissue damage at the site of infection in patients with cutaneous leishmaniasis.^23^ In this study, compound **1** did not modify production of this cytokine in infected and noninfected macrophages.

The cytokine IL-12 (p70) is composed of two subunits, p35 and p40 that are essential for resistance against *Leishmania*. The production of IL-12 is necessary for proper activation of macrophages as it induces the production of IFN-γ in NK cells. This cytokine is a potent stimulator of the effector functions of macrophages as it induces the expression of nitric oxide synthase (iNOS) to produce NO that helps to kill and control the proliferation of parasites. It also stimulates the differentiation of helper T cells (Th) into Th1 lymphocytes and inhibits T cell apoptosis.^24^ The promastigotes and amastigotes of *L. mexicana* modulate a negative production of this cytokine in macrophages, thus favoring their survival.^25^ In our results, compound **1** did not appear to affect the production of this cytokine in infected and noninfected macrophages.

The cytokine IL-10 has the function of inhibiting the production of IFN-γ which interferes on the production of NO in activated macrophages. It has been shown that IL-10-deficient BALB/c mice infected with *L. major* controlled disease progression and had relatively smaller lesions with 1000-fold fewer parasites inside them by the fifth week of infection, compared to normal BALB/c mice. The authors also showed that IL-10 produced by infected macrophages prevented macrophage activation and decreased their production of IL-12 and TNF-α.^26^ In contrast, C57BL/6 mice with IL-10 deficiency did not show higher resistance against *L. amazonensis* even when they developed a better Th1 response.^27^ Another study reported that IL-10 deficient BALB/c mice failed to control lesion development when infected with *L. mexicana*, but did demonstrate an increased ability to reduce parasite numbers in contrast to wild-type BALB/C mice.^28^ In humans, it has been observed that patients with LCL caused by *L. mexicana* who presented lesions with more than four months evolution time, presented a high expression of genes for TNF-α, IL-10 and TGF-β, whereas patients who presented a relapse after wound healing due to treatment with glucantime, showed an absence in IL-10 expression.^29^ This suggests that high levels of IL-10 are related to a greater development of *L. mexicana* disease, but low levels of this cytokine may favor wound healing. In this study, compound **1** was able to decrease IL-10 production in BMMs infected with *L. mexicana*, which may favor the resolution of LCL lesions.

**Figure 5:**
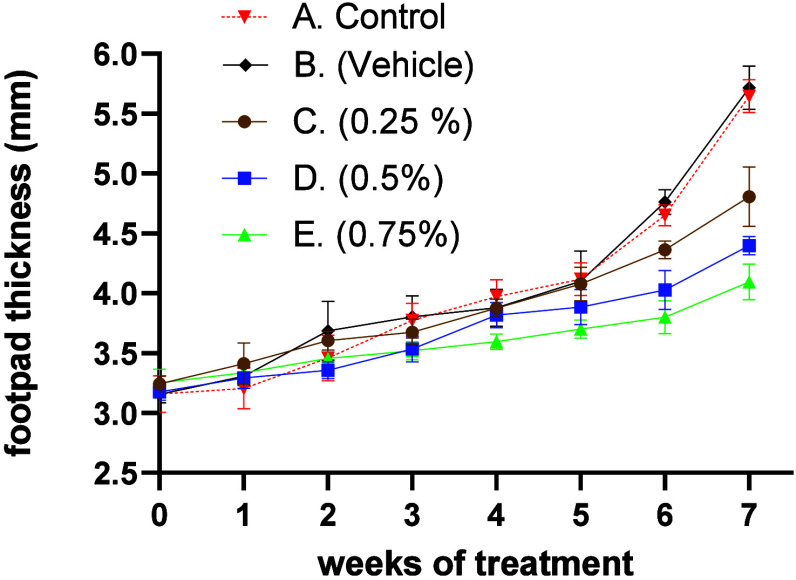
Topical effect of compound **1** on disease progression in C57BL/6 mice infected with *L. mexicana* for 7 weeks. (A) Control group of nontreated mice. (B) Mice treated with vehicle. (C) Mice treated with 0.25% compound **1**. (D) Mice treated with 0.5% compound **1**. (E) Mice treated with 0.75% compound **1**. Data show mean ± SD of two independent experiments (*n* = 3 mice).

To complete this study, the topical effect of compound **1** was analyzed in an *in vivo* model of C57BL/6 mice infected in the footpad with *L. mexicana* promastigotes, as described in the experimental section. The treatments with 0.5% and 0.75% of compound **1** showed effects on reducing the evolution of the disease between the fourth and fifth week, compared to nontreated mice, in which the size of the lesion increased steadily during the 7 week experiment. Likewise, treatments with 0.5% and 0.75% of compound **1** significantly decreased the thickness of the footpad by 50.2% and 62.6%, respectively, when compared to the lesion size in the group of mice without treatment at week 7 (*p* ≤ 0.05). The mice treated with the vehicle did not show a significant reduction in the thickness of the footpad (Figure 5).

**Figure 6:**
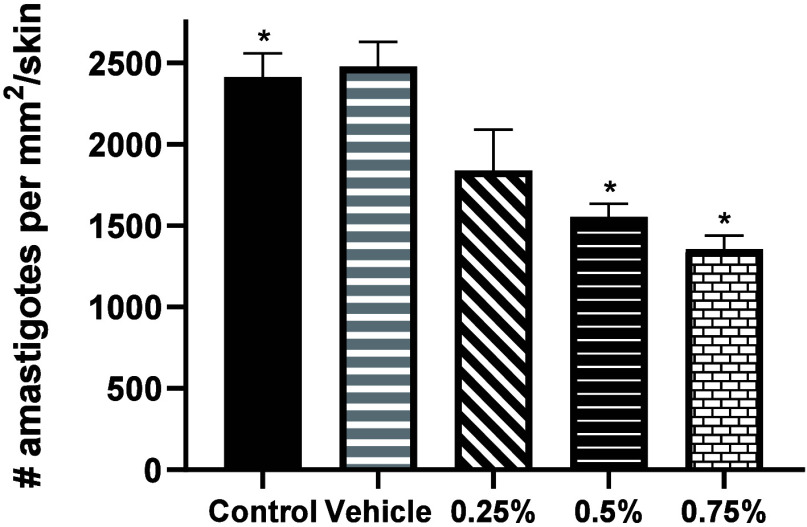
Effect of compound **1** on parasite load per mm^2^/skin in mice infected with *L. mexicana* after 7 weeks of treatment. Symbols indicate statistically significant differences compared to nontreated control mice (*p* ≤ 0.05). Bars represent mean + SD of 2 independent experiments (*n* = 3 mice).

At the end of the treatments, euthanasia was performed on all mice and the parasite load of the infected pads in each experimental group was determined by counting the number of amastigotes per mm^2^/skin. Treatments with 0.5% and 0.75% of compound **1** significantly reduced the number of amastigotes by 35.7% and 44.1%, respectively, compared to the group of mice without treatment (*p* ≤ 0.05). The vehicle had no effect on the reduction of the parasite load (Figure 6).

**Figure 7:**
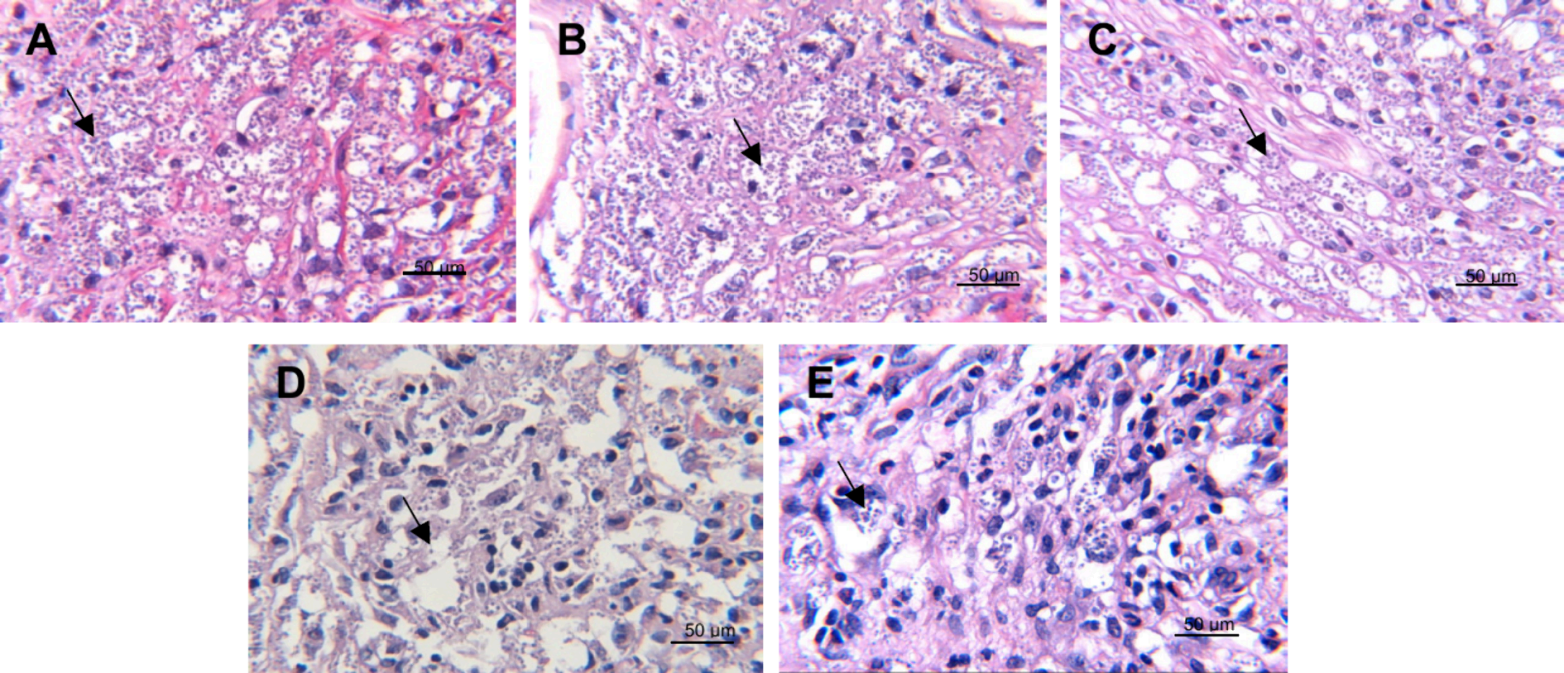
Amastigotes in footpads of C57BL/6 mice infected with *L. mexicana* and treated with compound **1**. The microphotographs (400×, H-E staining) show histological sections of skin with the presence of amastigotes. (A) Mice without treatment. (B) Mice treated with vehicle. (C) Mice treated with 0.25% compound **1**. (D) Mice treated with 0.5% compound **1**. (E) Mice treated with 0.75% compound **1**. Arrows point to some amastigotes. Scale bar = 50 μm.

When observing the parasite load in the microphotographs, it was possible to verify the decrease in the number of amastigotes in the treatments with 0.5% and 0.75% of compound **1**, compared to the control without treatment (Figure 7).

So far, our results indicate that compound **1** is well absorbed topically when applied with cream as vehicle. In addition, the lipophilic characteristic of this compound enables permeating cell membranes and increases its ability to cross the stratum corneum and reach the parasites.^30^

The present study complements the *in vitro* results previously reported for compound **1**, by demonstrating that its leishmanicidal effect is due to the generation of ROS that causes apoptosis in *Leishmania* parasites and shows that it has the potential to be used topically to counteract the LCL. These results are of interest, since there are still no fully effective topical treatments to control this disease. Furthermore, it remains to be determined whether this compound can be combined with other natural products or with any first-line drugs used for the treatment of LCL, in order to increase its efficacy in controlling the disease, with a lower cost and less invasive.

## Experimental Section

### General Experimental Procedures

The RPMI-1640 medium, 199 medium, 2,3-bis (2-methoxy-4-nitro-5-sulfophenyl)-2H-tetrazolium-5-carboxanilide (XTT) reagent, phenazine methosulfate (PMS) electron coupler, and dimethyl sulfoxide (DMSO) were acquired from Sigma-Aldrich (St Louis, MO, USA). The compound (3*S*)-16,17-didehydrofalcarinol (**1)** was isolated by chromatographic methods from *Tridax procumbens*. The purity and identification were established by GC-MS analysis and spectroscopic properties (Supporting Information: Figure S1-Figure S5).^31^ Fetal Bovine Serum (FBS) was purchased from Gibco Invitrogen Corporation (Carlsbad, CA, USA). Mineral oil, vegetable oils, cetyl alcohol, paraffin, lanolin and borax were obtained from Pharmacy Paris (Mexico City, Mexico).

### Parasites

*L. mexicana* strain (MHOM/MX/2011/Lacandona) promastigotes were previously obtained and cultured in 199 medium, supplemented with 10% heat-inactivated FBS with 1% antibiotics (100 U/mL of penicillin G and 100 mg/mL of streptomycin) for 5 days at 26 °C.^32^ Metacyclic promastigotes (day 5 of culture) were used for in *vitro* and *in vivo* experiments.

### Animals

Female C57BL/6 mice (8–10 weeks of age and a weight of 25–30 g) were bred and housed in the Animal Laboratory of the Experimental Medicine Unit of the UNAM School of Medicine. The experiments were conducted following the National Ethical Guidelines in Animal Health NOM-062-ZOO-1999 and the guidelines recommended for the care of animals by the Ethics Committee of the UNAM School of Medicine (CICUAL-024–2023). The animals were kept in pathogen-free cages and fed *ad libitum*.

### Bone Marrow-Derived Macrophages (BMMs)

BMMs were obtained from BALB/c mice. Briefly, the long bones (femur and tibia) of the mice were aseptically removed and the cells recovered with cold PBS. Cells (2 × 10^6^) were cultured in Petri dishes (Falcon, Corning, New York, NY, USA) with RPMI-1640 medium supplemented with 20% FBS and 20% supernatant from L929 fibroblast cultures, as a source of macrophage-stimulating factor (M-CSF). They were incubated at 37 °C with 5% CO_2_ for 7 days. Subsequently, adherent BMMs were collected, and purity was analyzed by flow cytometry (FACS Canto II, BD, Becton Dickson, San Jose, CA, USA). Anti-F4/80 mouse monoclonal antibody (mAb) staining (FITC) (Biolegend) showed that 98% of the cells were macrophages.

### Preparation of Topical Formulations

Topical formulations with (3*S*)-16,17-didehydrofalcarinol (**1**) were prepared as follows: vegetable oils, mineral oil, cetyl alcohol, paraffin, lanolin and propylparaben were placed in a glass cup (Part A). Distilled water, borax, and methylparaben were added to another glass (Part B). Parts A and B were heated in a water bath at 72 °C. Finally, both parts were mixed homogeneously until the consistency of a cream was obtained. Compound **1** was incorporated into this cream in concentrations of 0.25%, 0.5%, and 0.75%, respectively, for topical applications.

### Reactive Oxygen Species (ROS) Production

*L. mexicana* promastigotes (1 × 10^6^/mL) were incubated with 0.8 μM and 1.6 μM (0.2 μg/mL and 0.4 μg/mL, respectively) of compound **1** for 72 h at 26 °C. Nontreated parasites were used as viability control and DMSO 0.004% (0.04 μL/mL) was control vehicle. ROS production was measured with 2′,7′-dichlorodihydrofluorescein diacetate (H2CFDA), Sigma-Aldrich (St Louis, MO, USA), at a concentration of 10 μM for 30 min in PBS. The parasites were then washed and resuspended in PBS and analyzed by flow cytometry. The percentage of ROS in each sample was obtained with the FlowJo v.10 software (Treestar, Ashland, OR, USA) and the graph the GraphPad Prism 9 software. The results were compared with the nontreated control.

### Apoptosis Assay

*L. mexicana* promastigotes (1 × 10^6^/mL) were incubated in 4 mL tubes with 199 medium supplemented with 10% heat-inactivated FBS along with 1.6 μM (0.40 μg/mL) of compound **1** for 72 h at 26 °C. Nontreated promastigotes were used as growth control, and promastigotes incubated with 0.004% DMSO were used as vehicle control. After incubation, 2 × 10^6^ parasites of each condition were stained with APC Annexin V (Biolegend, San Diego, CA, USA) and 7-amino-actinomycin D (7-AAD) (Tonbo Bioscience) diluted 1:20 in Annexin V binding buffer (Tonbo Bioscience) for 15 min in the dark at room temperature. Samples were analyzed on the Cytek Aurora spectral cytometer (Cytek Biosciences, Fremont, CA, USA). The percentages of apoptosis and necrosis of each sample were analyzed by the FlowJo v.10 software and the graph the GraphPad Prism 9 software. The results were compared with the viability control.

### Cytotoxicity Assay

The cytotoxic effect of compound **1** was determined by the XTT colorimetric assay with modifications. This technique is based on the reduction of the salt 2,3-bis-(2-methoxy-4-nitro-5-sulfofenyl)-2H-tetrazolium-5-carboxinilide to orange crystals, by means of active respiration of the cells, an action that is accelerated with the help of the PMS electron coupler.^33^ Briefly, peritoneal macrophages isolated from BALB/c mice (1 × 10^5^) were incubated in a 96-well dish with RPMI-1640 medium (supplemented with 10% heat-inactivated FBS at 56 °C for 30 min and 1% of antibiotics (100 U/mL of penicillin G and 100 mg/mL of streptomycin) for 24 h at 37 °C and 5% CO_2_. Thereafter, macrophages were incubated with different concentrations of compound **1** (0.1, 0.2, 0.5, 1.0, 2.0, and 4.0 μM) for 48 h at 37 °C and 5% CO_2_. Nonstimulated macrophages were considered basal control, and cells incubated with 0.1 μL of DMSO were used as vehicle control. After adding 50 μL of XTT-PMS reagent (1 mg - 0.06 mg/mL), the samples were incubated during 4 h. Optical density (OD) of each sample was measured at 450 nm. The percentage of viable cells of each concentration was compared to the control without treatment and the CC_50_ was obtained using the GraphPad Prism 9 software. The selectivity index (SI) of compound **1** was obtained by dividing the CC_50_ of macrophages/CI_50_ of *L. mexicana* promastigotes reported in a previous study.^34^

### Nitric Oxide (NO) Production

NO production in *L mexicana*-infected BMMs incubated with compound **1** was determined by the Griess method.^35^ Briefly, macrophages were infected with *L. mexicana* promastigotes and treated with 0.40 μg/mL (1.6 μM) of compound **1** for 24 h in RPMI-1640 medium, supplemented with 10% FBS at 37 °C with 5% CO_2_. Nonstimulated macrophages (ø) were used as basal control and macrophages stimulated with 100 ng/mL lipopolysaccharide (LPS) from *Escherichia coli* 026:B6 (Sigma, St Lois, MO, USA) were used as a positive control. Macrophages incubated with *L. mexicana* (ø + *L.mex*) were used as infection control. Culture supernatants were collected and nitrites levels were quantified in 96-well plates using 100 μL per well of the Griess reagent (modified) (Sigma-Aldrich) and plates were incubated at room temperature in darkness for 10 min. A sodium nitrite solution in a range of 0–100 μM was used as the standard curve. Absorbance was measured on a MULTISKAN Sky (Thermo-scientific) reader at 540 nm. The nitrites concentration of each sample was calculated by regression analysis based on a standard curve. The detection limit of this assay ranged from 0.5 to 100 μM. Graphs were obtained using GraphPad Prism 9 software. The effect of compound **1** on nitrite production in BMMs infected with *L. mexicana* was compared with the infection control.

### Cytokine Production

BMMs differentiated from BALB/c mice (1 × 10^6^) were seeded in 1 mL of RPMI-1640 medium supplemented with 10% heat-inactivated FBS for 24 h at 37 °C and 5% CO_2_ in 24-well culture plates (Costar, Corning, New York, USA). After incubation time, macrophages were infected with 1 × 10^7^*L. mexicana* promastigotes (infection ratio 1:10), and after 24 h, they were treated with 0.40 μg/mL (1.6 μM) of compound **1**. Nontreated macrophages (ø) were used as a basal control. Macrophages stimulated with 100 ng/mL lipopolysaccharide (ø + LPS) were used as a positive control. Macrophages incubated with *L. mexicana* (ø + *L.mex*) were used as an infection control and macrophages stimulated with 1.6 μM of compound **1** (ø + 1.6 μM) as a negative control. All conditions were incubated again at 37 °C and 5% CO_2_ for 24 h. Subsequently, culture supernatants were collected and TNF-α, IL-10, and IL-12 (p70) concentrations were analyzed using the sandwich ELISA test. The samples were prepared in triplicate. Briefly, 96-well plates (Costar, Corning, NY, USA) were coated with mouse anti-TNF-α capture antibody (clone TN3–19.12, 1 μg/mL, BD Pharmigen), mouse anti-IL-10 capture antibody (clone JES5–2A5, 3 μg/mL, BD Pharmingen), and anti-IL-12 (p70) capture antibody (clone 9A5, 4 μg/mL, BD Pharmingen) in Na_2_HPO_4_ overnight at 4 °C and blocked with a 0.5% casein in PBS. Culture supernatants and recombinant cytokines were incubated for 2 h at room temperature. Subsequently, mouse anti-TNF-α (clone 516D1A1, 1 μg/mL, BD Pharmingen), mouse anti-IL-10 (clone SXC-1, 1.5 μg/mL, BD, Pharmingen), and mouse anti-IL-12 (p40/p70) (clone C17.8, 2 μg/mL) biotinylated antibodies were used in 1% BSA with Tween 20 for 1 h and incubated with an alkaline streptavidin-phosphate conjugate (Invitrogen) in 1% BSA with 0.5% Tween 20 for 30 min. Finally, the plates were developed using phosphatase substrate (0.005 mg/mL, Sigma-Aldrich). Absorbance was measured at 405 nm with a MULTISKAN Sky (Thermo-Scientific) reader and the TNF-α, IL-12 and IL-10 concentration of each sample was calculated by regression analysis based on a standard curve. The detection limit of this assay ranged from 15 to 2000 pg/mL. Graphs were obtained using GraphPad Prism 9 software. The results were compared against basal control and the infection control.

### Topical Application of Compound **1** Evaluation

C57BL/6 mice were inoculated in the left footpad with 2 × 10^5^ parasites/10 μL of PBS. After 5 week postinfection, five experimental groups (*n* = 3) were formed randomly as follows: (**A**) control without treatment, (**B**) cream only (vehicle), (**C**) cream with 0.25% compound **1**, (**D**) cream with 0.5% compound **1**, (**E**) cream with 0.75% compound **1**. Treatments (20 mg/day) were applied topically for 7 weeks. The effect of each treatment on the evolution of the disease was evaluated by measuring the thickness (mm) of the infected footpads by photographs taken at weekly intervals, until the end of the experiment. The results were compared with the nontreated control group. After completing all the experiments, the mice were anesthetized by exposure to carbon dioxide (CO_2_) and euthanized by cervical dislocation.

### Parasite Load Evaluation

After euthanasia, infected footpads were cut and fixed with 10% formol. They were embedded in paraffin and 3 μm thick sections were stained with hematoxylin/eosin (HE). Eight microphotographs of each sample were taken with an Axio-Imager.M1 microscope and camera (AxioCam MRc5), corresponding to 1 mm^2^/skin. The parasite load of the different experimental groups (# amastigotes/mm^2^ of skin) was quantified. The results were compared with the nontreated control.

### Statistical Analysis

The results were analyzed using the nonparametric Mann–Whitney U test for comparison between experimental groups. CC_50_ was obtained with GraphPad Prism 9 software. In all statistical analyses, a significant difference was considered with a *p* ≤ 0.05.

## Supporting Information Available

GC-FID chromatogram, infrared spectrum, ^1^H-RMN and ^13^C-RMN spectra, and low-resolution GC-EM spectrum of (3*S*)-16,17-didehydrofalcarinol
  (PDF)
