# Supplementary material for: Topical Application of Oxylipin (3S)-16,17-Didehydrofalcarinol in Mice Infected with Leishmania mexicana: A Possible Treatment for Localized Cutaneous Leishmaniasis
Source: J Nat Prod. 2025 Apr 3;88(4):959–66. doi: 10.1021/acs.jnatprod.4c01411 (PMC12038837; doi:10.1021/acs.jnatprod.4c01411)
Supplement: Supplementary file 1 — np4c01411_si_001.pdf [file np4c01411_si_001.pdf]

Supporting Information

Topical application of oxylipin (3*S*)-16,17-didehydrofalcarinol in mice infected with *Leishmania mexicana*: a possible treatment for localized cutaneous leishmaniasis

Ana G. Carrillo-Ake[1], José Delgado-Domínguez[1], Rocely Buenaventura Cervantes-Sarabia[1], Adriana Ruiz-Remigio[1], Jaime Zamora-Chimal[1], Norma Salaiza-Suazo[1], Luis W. Torres-Tapia[2], Sergio R. Peraza-Sánchez[2*] e Ingeborg Becker[1*].

[1]Unidad de Medicina Experimental, Facultad de Medicina, Universidad Nacional Autónoma de México (UNAM), Hospital General de México Dr. Balmis 148, Ciudad de México C.P. 06720, México.

[2]Centro de Investigación Científica de Yucatán (CICY), Unidad de Biotecnología, Calle 43 #130, Col. Chuburná de Hidalgo, 97205, Mérida, Yucatán, México.

* speraza@cicy.mx (S.R.P.S) and becker@unam.mx (I.B.)

Page	Contents
S2	Figure S1. GC-FID Chromatogram of the (3*S*)-16,17-didehydrofalcarinol isolated from roots of *T. procumbens* and pelargonic acid vanillylamide used as internal standard.
S3	Figure S2. Infrared spectrum of (3*S*)-16,17-didehydrofalcarinol.
S4	Figure S3. ^{1}H-NMR spectrum of (3*S*)-16,17-didehydrofalcarinol.
S5	Figure S4. ^{13}C-RMN spectrum of (3*S*)-16,17-didehydrofalcarinol.
S6	Figure S5. Low resolution GC-EM spectrum of (3*S*)-16,17-didehydrofalcarinol.

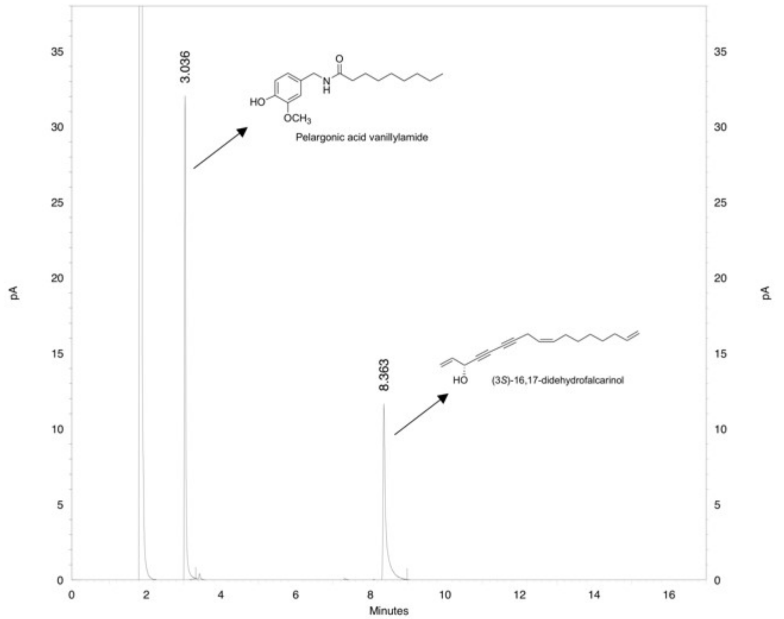

Figure S1. GC-FID Chromatogram of the (3*S*)-16,17-didehydrofalcarinol isolated from roots of *T. procumbens* and pelargonic acid vanillylamide used as internal standard.

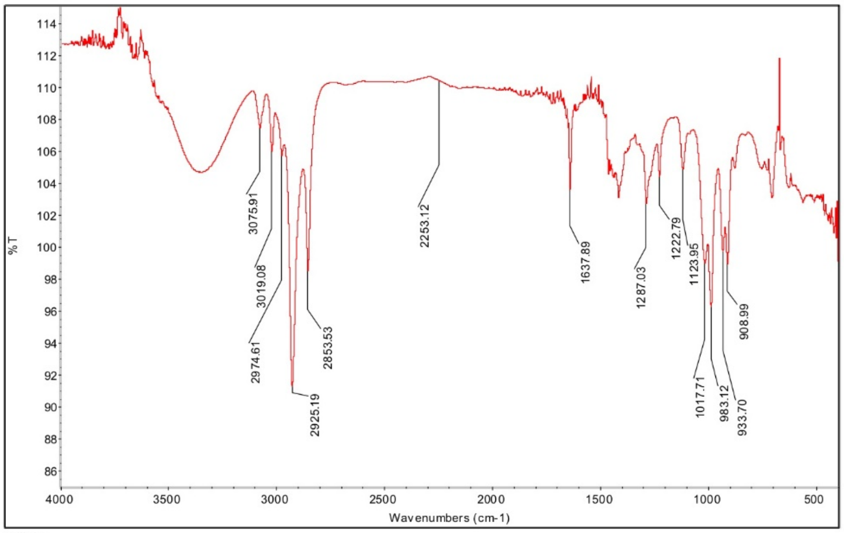

Figure S2. Infrared spectrum of (3*S*)-16,17-didehydrofalcarinol.

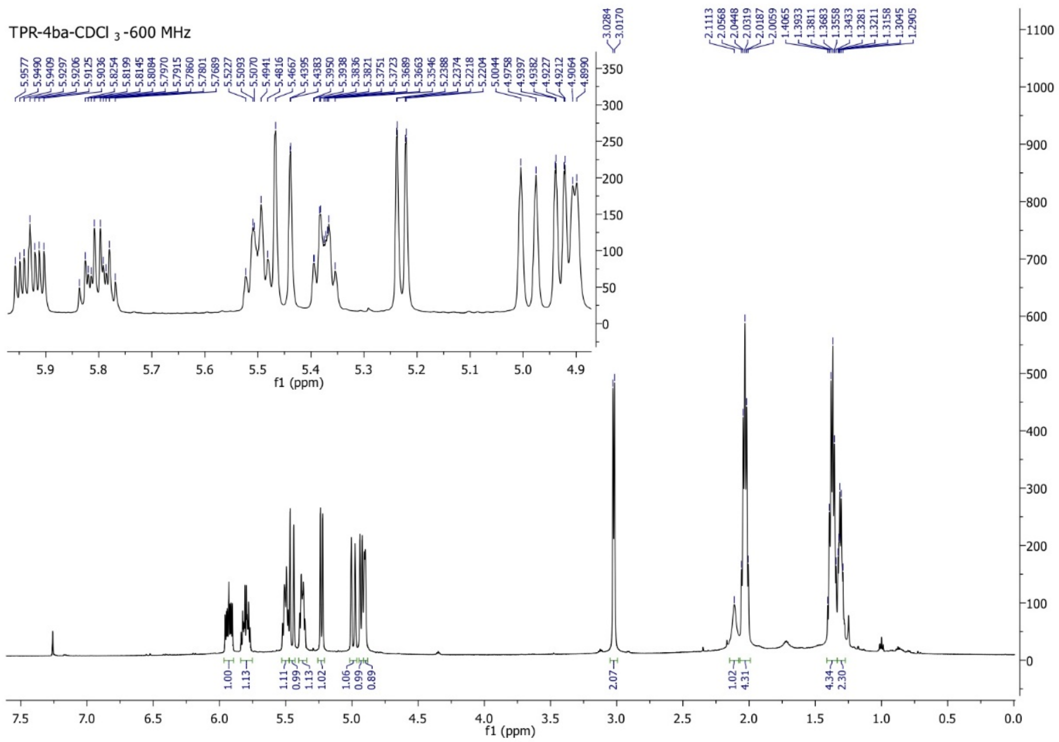

Figure S3. ^{1}H-NMR spectrum of (3*S*)-16,17-didehydrofalcarinol.

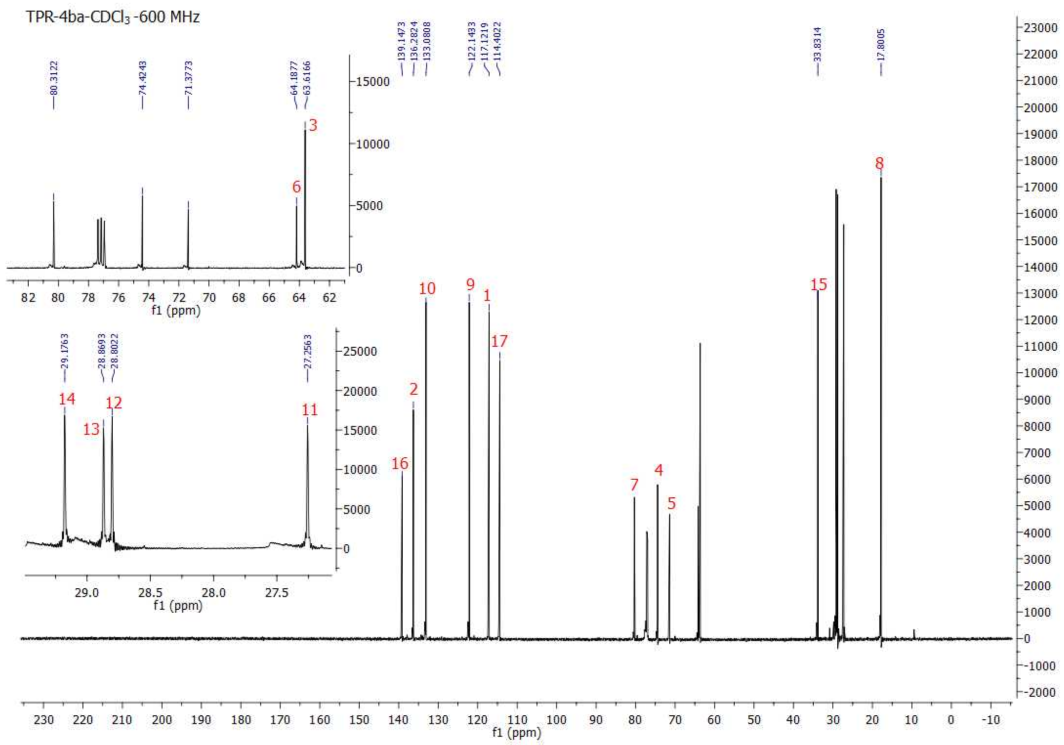

Figure S4. ^{13}C-RMN spectrum of (3*S*)-16,17-didehydrofalcarinol.

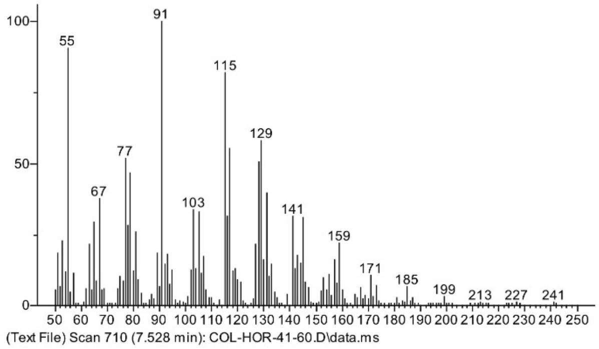

Name: Scan 710 (7.528 min): TPR-4a1\data.ms

MW: N/A ID#: 63 DB: Text File Comment: TPR-4a1

10 largest peaks:

91	999	115	818	117	552	128	504	131	394
55	903	129	579	77	516	79	465	67	376

156 m/z Values and Intensities:

50	52	83	40	117	552	152	48	186	15
51	181	84	3	118	118	153	96	187	25
52	63	85	6	119	128	154	53	188	5
53	224	86	19	120	90	155	108	189	1
54	117	87	37	121	82	156	32	193	1
55	903	88	23	122	15	157	158	194	2
56	45	89	182	123	5	158	75	195	6
57	113	90	65	125	2	159	217	196	2
58	6	91	999	126	22	160	51	197	4
59	2	92	143	127	213	161	20	198	3
61	10	93	177	128	504	162	3	199	28
62	55	94	71	129	579	163	1	200	6
63	212	95	124	130	158	165	36	201	6
64	54	96	17	131	394	166	27	202	1
65	293	97	6	132	101	167	60	209	2
66	85	98	13	133	144	168	23	211	4
67	376	99	9	134	45	169	34	212	1
68	53	100	4	135	27	170	20	213	11
69	58	101	31	136	5	171	102	214	2
70	7	102	122	137	1	172	31	215	6
71	4	103	335	139	36	173	69	216	1
72	2	104	127	141	310	174	13	223	1
73	4	105	329	142	127	175	5	224	1
74	57	106	111	143	173	176	1	225	1
75	100	107	169	144	146	178	3	227	8
76	83	108	53	145	309	179	7	228	1
77	516	109	25	146	80	180	6	241	8
78	282	110	24	147	60	181	26	242	2
79	465	111	5	148	11	182	7		
80	121	113	16	149	4	183	15		
81	255	115	818	150	2	184	9		
82	88	116	313	151	9	185	64		

Figure S5. Low resolution GC-EM spectrum of (3*S*)-16,17-didehydrofalcarinol.
